# Supplementary material for: Cardiomyocyte-Specific Deletion of Orai1 Reveals Its Protective Role in Angiotensin-II-Induced Pathological Cardiac Remodeling
Source: Cells. 2020 Apr 28;9(5):1092. doi: 10.3390/cells9051092 (PMC7290784; doi:10.3390/cells9051092)
Supplement: Supplementary file 1 [file cells-09-01092-s001.pdf]

SUPPLEMENTARY MATERIAL

Cardiomyocyte-specific deletion of Orai1 reveals its protective role in Angiotensin-II-induced pathological cardiac remodeling

Sebastian Segin[1,2], Michael Berlin[1,2], Christin Richter[1], Rebekka Medert[1,2] Veit Flockerzi[3], Paul Worley[4], Marc Freichel[1,2] and Juan E. Camacho Londoño[1,2]

1. Pharmakologisches Institut, Ruprecht-Karls-Universität Heidelberg, 69120 Heidelberg, Germany.
2. DZHK (German Centre for Cardiovascular Research), partner site Heidelberg/Mannheim, Germany.
3. Experimentelle und Klinische Pharmakologie und Toxikologie, Universität des Saarlandes, 66421 Homburg, Germany.
4. The Solomon H. Snyder Department of Neuroscience, Johns Hopkins University, School of Medicine, MD 21205 Baltimore, USA.

Correspondence to: Juan E. Camacho Londoño, INF-366, D-69120 Heidelberg, Germany, Tel.: +49-6221-54-86863, Fax: +49-6221-54-8644
e-mail: juan.londono@pharma.uni-heidelberg.de

Figure S1. Induction of cellular hypertrophy in neonatal cardiomyocytes from wild type mice.
Figure S2. Efficiency and specificity of Cre-expression under the α-MHC promotor.
Table S1. Allometric analysis of Orai1$^{fx/fx}\alpha$MHC/Cre^{pos} mice.
Table S2. Allometric analysis of Orai1-WT and Orai1$^{CM\text{-}KO}$ mice after angiotensin II treatment.
Table S3. Primers used for qPCR analysis.

Supplementary Discussion. *Gene deletion of Orai1 and controls for Tamoxifen treatment or Cre expression.*

Supplementary Figures

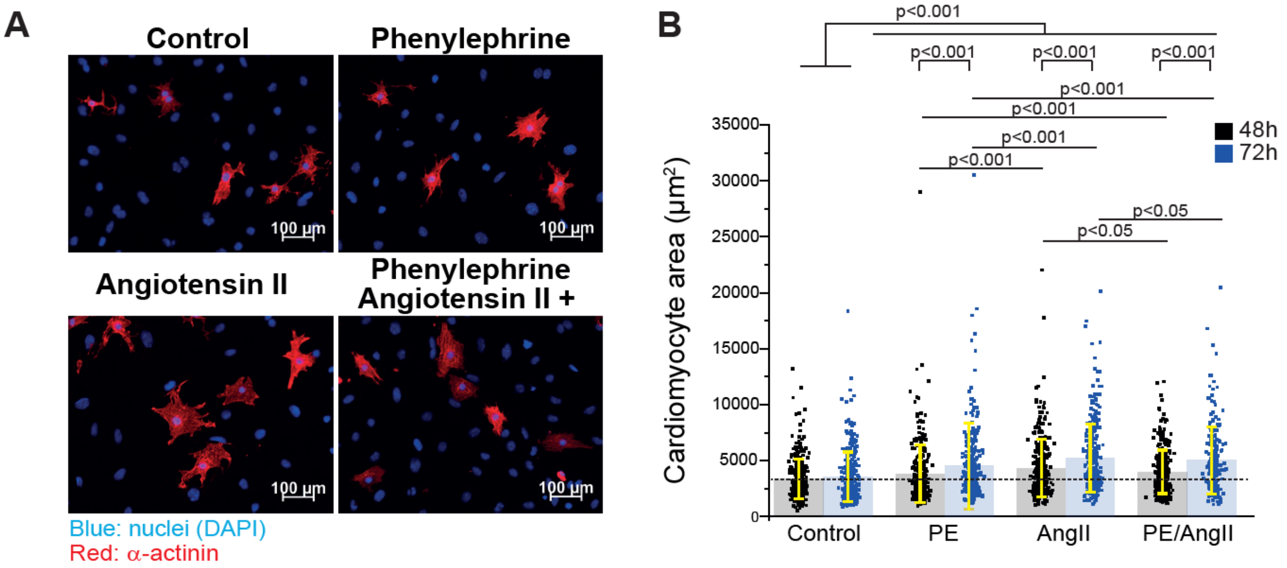

Figure S1: Induction of cellular hypertrophy in neonatal cardiomyocytes from wild type mice. (A) Representative images from α-actinin stained (red) cells after 72h of neurohumoral stimulation with angiotensin II (AngII) 100nM, Phenylephrine (PE) 50µM or both together. **(B)** Statistical analysis of cardiomyocyte cell area change after 48h and 72h of neurohumoral treatment. n=221-322 cardiomyocytes/group isolated from C57BL6/N mice. Bars represent mean +/- SD. Statistical comparison done with Kruskall-Wallis and Dunn's Multiple comparison tests.

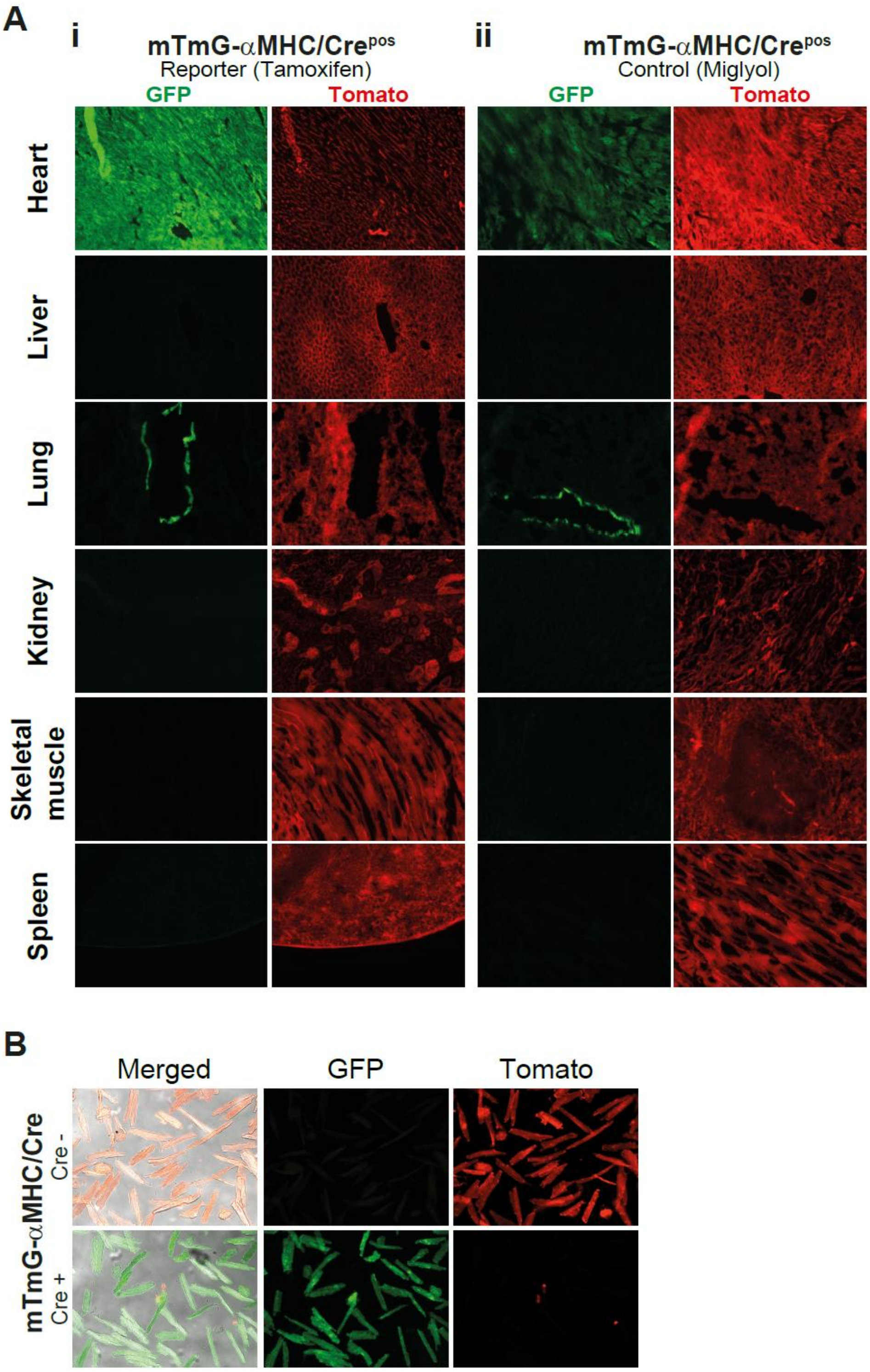

Figure S2: Efficiency and specificity of Cre-expression under the α-MHC promotor. (A) Representative pictures of tissue sections from hearts and other organs isolated from mTmG-αMHC/Cre+ mice treated either with Tamoxifen (i) or with Miglyol (ii) are depicted. GFP and Tomato fluorescence were analyzed to determine Cre activity 3 weeks after last Tamoxifen or Miglyol injection. 4≥n mice group were analyzed. **(B)** Representative images of adult cardiomyocytes isolated from the mTmG reporter mice expressing Cre under the αMHC promotor following treatment with Tamoxifen. Cre activity was evaluated by detection of GFP fluorescence (green) and compared Cre^{neg} cells in which only Tomato (red) was detected (n≥ 6/group).

Supplementary Tables

Table S1. Allometric analysis of Orai1-WT and Orai1$^{CM\text{-}KO}$ mice. Here Orai1$^{flox/flox}$/αMHC/Cre^{Pos} mice treated either with Miglyol (Orai1-WT) or with tamoxifen (Orai1$^{CM\text{-}KO}$) were compared. Three weeks after end of tamoxifen or Miglyol treatment mice were analyzed.

	**Orai1-WT**	**Orai1$^{CM\text{-}KO}$**	**p-value**
	n=18	n=14	
**Age (weeks)**	11.33 (±0.69)	10.93 (±0.73)	0.118
**Tibia length (mm)**	18.17 (±0.66)	18.26 (±0.54)	0.659
**Body weight (g)**	30.22 (±2.81)	27.72 (±3.96)	**0.046**
**Heart weight (mg)**	132.82 (±12.53)	129.09 (±17.04)	0.481
**Lung weight (mg)**	128.88 (±12.15)	128.28 (±16.70)	0.907
**Water content lung (%)**	74.69 (±2.96)	76.82 (±3.21)	0.061
**Heart weight (mg) / Body weight (g)**	4.40 (±0.29)	4.67 (±0.29)	**0.014**
**Heart weight (mg) / Tibia length (mm)**	7.31 (±0.56)	7.07 (±0.92)	0.374
**Lung weight (mg)**	128.88 (±12.15)	128.28 (±16.70)	0.907
**Lung weight (mg) / tibia length (mm)**	7.10 (±0.63)	7.04 (±1.01)	0.852
**Liver weight (mg)**	1461.11(±201.35)	1250.51 (±236.84)	**0.011**
**Water content liver (%)**	66.76 (±1.35)	67.88 (±0.99)	**0.015**
**Liver weight (mg) / tibia length (mm)**	80.80 (±11.00)	68.50 (±13.07)	**<0.01**

n= Number of mice. Values are given as mean ± SD

Table S2: Allometric analysis of Orai1-WT and Orai1$^{CM\text{-}KO}$ mice after angiotensin II treatment. Here Orai1$^{fx/fx}$$\alpha$MHC/Cre^{neg} or Orai1$^{fx/fx}$$\alpha$MHC/Cre^{Pos} mice were treated with tamoxifen to generate the corresponding Orai1-WT or cardiomyocyte-specific deficient Orai1 mice (Orai1$^{CM\text{-}KO}$). After three weeks of tamoxifen treatment end, mice were implanted with osmotic minipumps (AngII or NaCl) and 14 days after pump implantation mice were analyzed.

	**Orai1-WT**		**p-1**	**Orai1$^{CM\text{-}KO}$**		**p-2**	**p-3**
	**NaCl 0.9%**	**AngII**		**NaCl 0.9%**	**AngII**		
	n=18	n=14		n=18	n=20		
**Age (weeks)**	13.83 (±1.54)	14.57 (±0.94)	0.426	14.39 (±1.46)	14.60 (±1.31)	0.963	1
**Body weight (g)**	30.73 (±3.07)	25.91 (±2.35)	**<0.001**	30.65 (±3.72)	26.16 (±2.18)	**<0.001**	1
**Tibia length (mm)**	18.02 (±0.64)	17.76 (±0.80)	1	18.09 (±0.67)	18.00 (±0.78)	1	1
**Heart weight (mg)**	136.31 (±20.37)	173.7 (±34.23)	**<0.001**	140.69 (±22.09)	186.66 (±23.64)	**<0.001**	0.848
**Heart weight (mg) / Body weight (g)**	4.42 (±0.31)	6.66 (±0.81)	**<0.001**	4.58 (±0.40)	7.14 (±0.78)	**<0.001**	0.151
**Heart weight (mg) / Tibia length (mm)**	7.57 (±1.17)	9.83 (±2.18)	**<0.001**	7.80 (±1.34)	10.40 (±1.45)	**<0.001**	1
**Lung weight (mg)**	146.07 (±25.68)	143.41 (±27.78)	1	138.62 (±17.14)	160.16 (±66.61)	0.660	1
**Water content lung (%)**	79.56 (±6.41)	75.48 (±1.55)	**0.023**	74.55 (±2.65)	76.64 (±1.66)	0.619	1
**Lung weight (mg) / body weight (g)**	4.79 (±0.93)	5.50 (±0.68)	1	4.54 (±0.42)	6.24 (±3.22)	**0.034**	1
**Lung weight (mg) / tibia length (mm)**	8.14 (±1.64)	8.11 (±1.73)	1	7.67 (±1.00)	8.96 (±4.01)	0.686	1
**Liver weight (mg)**	1572.03 (±250.95)	1303.15 (±128.79)	**<0.01**	1406.77 (±285.70)	1262.26 (±184.00)	0.308	1
**Water content liver (%)**	66.35 (±1.55)	69.09 (±1.43)	**<0.001**	66.80 (±1.15)	68.94 (±1.25)	**<0.001**	1
**Liver weight (mg) / body weight (g)**	50.96 (±3.99)	50.40 (±4.07)	1	46.80 (±5.51)	49.33 (±6.24)	0.808	1
**Liver weight (mg) / tibia length (mm)**	87.28 (±13.30)	73.50 (±7.94)	**0.018**	79.75 (±16.39)	71.63 (±10.08)	0.299	1

AngII: Angiotensin II, n= Number of mice, p-1: p-value of comparison between WT-NaCl and WT-AngII, p-2: p-value of comparison between Orai1$^{CM\text{-}KO}$-NaCl and Orai1$^{CM\text{-}KO}$-AngII, p-3: p-value of comparison between WT-AngII and Orai1$^{CM\text{-}KO}$-AngII. Values are given as mean ± SD

Table S3: Primers used for qPCR analysis. Primers sequences and probe number for from the Universal Probe Library (Roche)

Primer name	primer sequence	probe number
Orai1 fw	caaccacagcgacagcag	46
Orai1 rev	gataaaaaccaggccacagg	
Orai2 fw	cagaggtgtcagcccatgt	51
Orai2 rev	caccaactcctgacaaagctg	
Orai3 fw	gctacttgacaagggttggtg	7
Orai3 rev	gttctgcattctaagggctga	
Saraf fw	tactccgaccgctacacca	75
Saraf rev	tgcctccaacacacttcaac	
Stim1 fw	gctgaggaggataatggttcc	94
Stim1 rev	acttcttcctgcctggactg	
Stim2 fw	acctcctctctgtactctgacca	9
Stim2 rev	cagcaatagggtagggtgga	
TGFb1 fw	cagagacgtggggacttctt	16
TGFb1 rev	gagcagggctgtctggagt	
TGFb2 fw	cttaccctaagcgagaaagtgc	31
TGFb2 rev	gcacccttccctagcttctc	
TGFb3 fw	aaagggaaagcatgaatgga	50
TGFb3 rev	gctaaaggtgggggcagt	
Col I fw	ggtctagacatgttcagctttgtg	92
Col I rev	ggcagtggcccctaagag	
Col III fw	tcccctggaatctgtgaatc	49
Col III rev	tgagtcgaattggggagaat	
CTGF fw	cgccaagcctgtcaagtt	71
CTGF rev	ccgcagaacttagccctgta	
A-SMA fw	ccgccatgtatgtggctatt	56
A-SMA rev	cagttgtacgtccagaggcata	
ANP fw	tgatggatttcaagaacctgct	25
ANP rev	cctcatcttctaccggcatc	
BNP fw	cttctgcggcatggatct	56
BNP rev	cagcggtgacagataaaggaa	
b-MHC fw	gctcttgtgctacccagctc	1
b-MHC rev	ttccacctaaagggctgttg	
Skel a-a fw	aatgagcgtttccgttgc	11
Skel a-a rev	cgcagactccataccgataaa	
Rcan1 fw	tttatatgccatctccaagctg	69
Rcan1 rev	tgggtctcttcattctctcca	
Myoz2 fw	gtgccgtcatattccacctt	69
Myoz2 rev	tttgcttcaccatggcacta	
Ppp3ca fw	tacaatcttctcggcaccaa	32
Ppp3ca fw	gaaatttgggagccagtacg	
Xirp2 fw	aacctgcaacgccttttct	108
Xirp2 fw	tcttgactgctgtggattgc	
Mef2a fw	cagcagcaccatctaggaca	19
Mef2a fw	ggacaaatttgaaccctgaga	
TRPC1 fw	ctgaaggatgtgcgagaggt	63
TRPC1 rev	cacgccagcaagaaaagc	
TRPC3 fw	ggtgaactgaaagaaatcaagca	19
TRPC3 rev	cgtcgcttggctcttatctt	
TRPC4 fw	aaacttttggttcagaaaggtgtc	104
TRPC4 rev2	acagttacagcggacctcgt	
TRPC6 fw	aggcaaaaggttagcgacaa	20
TRPC6 rev	ggcataaaagtcatcttgctgaa	
H3F3A fw	gccatctttcaattgtgttcg	19
H3F3A rev	agccatggtaaggacacctc	
AIP fw	accagtcatccaccaagagg	66
AIP rev	aggcgatggcgtcatagta	
CXCC1 fw	tagtgccgaccgctgact	26
CXCC1 rev	ggcctctcccctaactgaat	

Fw: Forward, rev: reverse.

Supplementary Discussion

Gene deletion of Orai1 and controls for Tamoxifen treatment or Cre expression. Using the αMHC/CreERT2 mice [1] crossed to mTmG reporter mice [2] we showed that we can induce highly efficient Cre-mediated recombination in adult cardiomyocytes in more than 99% of the isolated cardiomyocytes based on the detection of the Cre-dependent GFP fluorescence at a given time point by Tamoxifen injection. Based on our analysis of multiple organs, we corroborate the specificity of Cre-mediated recombination in cardiomyocytes except for a few cells in larger vessels of the lung that was not observed before with the Rosa26Lacz reporter line [1]. Thus, it can be assumed that with the use of this αMHC/CreERT2 mouse in combination with $Orai1^{flox/flox}$ mice [3], a complete abrogation of Orai1 proteins in adult cardiomyocytes can be achieved. Indeed, we observe by qPCR a reduction in *Orai1* expression by 52±13% in heart samples and 81.4±5.4% in cardiomyocytes isolated by Langendorff perfusion from $Orai1^{CM\text{-}KO}$ mice. The remaining expression most likely reflects the contribution of non-myocytes as Orai1 expression in α-Actinin negative cells isolated from embryonic heart was also found in our study.

In one experimental group used for allometric analysis of Orai1 deficient mice we compared $Orai1^{flox/flox}$/αMHC/Cre^{pos} mice treated with either Tamoxifen or Miglyol. We observed that the liver weight and body weight in $Orai1^{CM\text{-}KO}$ mice (Tamoxifen-treated mice) were significantly reduced in comparison to the corresponding control group (Miglyol). The difference in body weight most likely explains the difference in heart/weight to body weight index between both groups and it is attributable to the effect of the Tamoxifen treatment, rather to the Orai1-deletion since comparison of $Orai1^{CM\text{-}KO}$ mice ($Orai1^{flox/flox}$/αMHC-Cre^{pos} mice treated with Tamoxifen) to control $Orai1^{flox/flox}$/αMHC-Cre^{neg} mice treated with Tamoxifen revealed no differences regarding the Orai1 deletion in cardiomyocytes on liver weight or body weight. In line with this, the effect of tamoxifen on body weight has been reported and probably it changes depending on the concentration, mouse strain, type of diet, among others [4, 5].

References

[1] M. Takefuji, A. Wirth, M. Lukasova, S. Takefuji, T. Boettger, T. Braun, T. Althoff, S. Offermanns, N. Wettschureck, G(13)-mediated signaling pathway is required for pressure overload-induced cardiac remodeling and heart failure, Circulation 126(16) (2012) 1972-82 DOI: 10.1161/CIRCULATIONAHA.112.109256.

[2] M.D. Muzumdar, B. Tasic, K. Miyamichi, L. Li, L. Luo, A global double-fluorescent Cre reporter mouse, Genesis 45(9) (2007) 593-605 DOI: 10.1002/dvg.20335.

[3] M. Ahuja, D.M. Schwartz, M. Tandon, A. Son, M. Zeng, W. Swaim, M. Eckhaus, V. Hoffman, Y. Cui, B. Xiao, P.F. Worley, S. Muallem, Orai1-Mediated Antimicrobial Secretion from Pancreatic Acini Shapes the Gut Microbiome and Regulates Gut Innate Immunity, Cell Metab 25(3) (2017) 635-646 DOI: 10.1016/j.cmet.2017.02.007.

[4] F.F. Gao, J.W. Lv, Y. Wang, R. Fan, Q. Li, Z. Zhang, L. Wei, Tamoxifen induces hepatotoxicity and changes to hepatocyte morphology at the early stage of endocrinotherapy in mice, Biomed Rep 4(1) (2016) 102-106 DOI: 10.3892/br.2015.536.

[5] Z. Liu, Y. Cheng, Y. Luan, W. Zhong, H. Lai, H. Wang, H. Yu, Y. Yang, N. Feng, F. Yuan, R. Huang, Z. He, F. Zhang, M. Yan, H. Yin, F. Guo, Q. Zhai, Short-term tamoxifen treatment has long-term effects on metabolism in high-fat diet-fed mice with involvement of Nmnat2 in POMC neurons, FEBS Lett 592(19) (2018) 3305-3316 DOI: 10.1002/1873-3468.13240.
